# Prevalence and associated factors of anemia in a Russian population: the Ural eye and medical study

**DOI:** 10.1186/s12889-019-7016-6

**Published:** 2019-06-14

**Authors:** Mukharram M. Bikbov, Gyulli M. Kazakbaeva, Rinat M. Zainullin, Venera F. Salavatova, Timur R. Gilmanshin, Dilya F. Yakupova, Yulia V. Uzianbaeva, Inga I. Arslangareeva, Songhomitra Panda-Jonas, Svetlana R. Mukhamadieva, Renat I. Khikmatullin, Said K. Aminev, Ildar F. Nuriev, Artur F. Zaynetdinov, Jost B. Jonas

**Affiliations:** 1 Ufa Eye Research Institute, 90 Pushkin Street, Ufa, 450077 Bashkortostan Russia; 2 Department of Ophthalmology, Medical Faculty Mannheim of the Ruprecht-Karls-University of Heidelberg, Theodor-Kutzerufer 1, 68167 Mannheim, Germany

**Keywords:** Anemia, Russia, Population-based study, Ural eye and medical study

## Abstract

**Background:**

Although anemia is one of the leading causes of the global burden of disease, information about its prevalence in Russia is mostly missing. We therefore assessed its prevalence and associated factors in a Russian population.

**Methods:**

The population-based Ural Eye and Medical Study included 5899 (80.5%) out of 7328 eligible individuals (mean age:59.0 ± 10.7 years;range:40–94 years) who underwent a standardized interview and detailed general examination. The definition of anemia was based on the hemoglobin concentration (definition #1:hemoglobin concentration < 140 g/L in men,< 130 g/L in women; definition #2:hemoglobin concentration < 130 g/L in men,< 120 g/L in women [World Health Organization definition]).

**Results:**

Higher hemoglobin concentration (mean:142.6 ± 14.8 g/L; range:80-171 g/L) was associated (multivariable analysis) with male gender (*P* < 0.001; standardized regression coefficient beta:-0.20), higher waist-hip circumference ratio (*P* < 0.001;beta:0.05), higher prevalence of car ownership (*P* < 0.001;beta:0.05), higher blood concentrations of bilirubin (*P* < 0.001;beta:0.05) and triglycerides (*P* < 0.001;beta:0.06), lower erythrocyte sedimentation rate (*P* < 0.001;beta:-0.32), and shorter blood clotting time (*P* < 0.001;beta:-0.39). Using definition #1 and #2, anemia was detected in 1385 individuals (23.6%;95% confidence interval CI)CI:22.5,24.7) and in 453 individuals (7.7%;95%CI:7.0,8.4), respectively. Prevalence of moderate anemia (hemoglobin concenttration:110 g/L-80 g/L), detected in 165 individuals (2.8%;95%CI:2.4,3.2), increased with younger age (*P* = 0.008;odds ratio (OR):0.98;95%CI:0.96,0.99), female gender (*P* < 0.001;OR:2.52;95%CI:1.47,4.33), higher erythrocyte sedimentation rate (*P* < 0.001;OR:1.08;95%CI:1.06,1.09), longer blood clotting time (*P* < 0.001;OR:8.56;95%CI:5.68,12.9), and marginally significantly, with a lower waist-hip circumference ratio (*P* = 0.058;OR:0.13;95%CI:0.02,1.07). In women, it was significantly (*P* < 0.001) higher before menopause (8.8%;95%CI:6.4,11.1) than after menopause (3.5%;95%CI:2.8,4.3).

**Conclusions:**

In this Russian population as compared to populations from countries with a similar socio-demographic index, anemia prevalence was relatively low. As in other populations, higher anemia prevalence was strongly and inversely associated with menopause, and to a minor degree, with lower waist-hip circumference ratio and lower socio-economic background.

## Background

The Global Burden of Diseases, Injuries and Risk Factors (GBD) Study estimated in 2010 that the global prevalence of anemia was 32.9%, and that anemia caused 68.4 million YLDs (years lived with disability) or 8.8% of all YLDs [1, 2]. That figure was higher than the figures for major depression (63.2 million YLD; 8.2%), chronic respiratory diseases (49.3 million YLDs, 6.3%) and the whole of all injuries (47.2 million YLDs; 6.3%). The anemia-associated YLDs increased in all ages between 1990 and 2010, driven by a combination of increased prevalence and population growth in the young age groups. In adulthood, the increase in the anemia-related YLDs was attributed to population growth and enhanced survival of chronic diseases associated with anemia, such as chronic kidney disease. In elderly groups, the increase in the anemia-associated YLDs was mainly due to population aging while the anemia prevalence in the elderly age groups decreased from all causes except for malaria. In most regions and age groups, anemia prevalence was higher in females than in males [1–3].

Despite the general importance of anemia for public health and although Russia is by area the largest, and by population one of the most populous countries worldwide, information about the prevalence of anemia in Russia and factors associated with the occurrence of anemia in Russia has been mostly missing so far. We therefore conducted this study to assess the prevalence of anemia in a population in Russia and to explore its associations with other factors [4, 5].

## Methods

Designed as a population-based investigation, the Ural Eye and Medical Study (UEMS) was carried out in the Ufa (urban region of Kirovskii), the capital of the Russian Republic of Bashkortostan, and in villages of the Karmaskalinsky District located in a distance of 65 km from Ufa [6]. Bashkortostan (total population: 4.07 million) is located in the west of the southern Ural Mountains. The population of Ufa with 1.1 million inhabitants includes Russians, Tatars, Bashkirs, Ukrainians and other ethnicities. While there were no exclusion criteria, the only inclusion criteria for the participation in the study were an age of 40 or more years and living in the study region.

The series of examinations started with a standardized interview which was performed by trained social workers. It consisted of more than 250 questions about socioeconomic variables (e.g., educational level), life style including diet, smoking and alcohol consumption, depression and suicidal ideation, physical activity, and medical history including known diagnosis and therapy of major systemic diseases including presence or history of previous iron-deficiency related anemia. The data were reported using the Guidelines for Accurate and Transparent Health Estimates Reporting: the GATHER statement guidelines [7].

The physical examinations included measurement of blood pressure, pulse rate and body height, body weight and hip and waist circumference. Using a dynamometer, we determined the handgrip strength. We measured hearing loss by hearing loss-related questions in the interview and by conducting Rinne’s test and Weber’s test. The assessment of blood samples, which were taken under fasting conditions, included a complete blood cell count and determinations of the serum concentrations of glucose, blood lipids, C-reactive protein, bilirubin, urea, creatinine, and other substances. The pulmonary function was tested by spirometry. Diabetes mellitus was defined by a glucose concentration of ≥7.0 mmol/L or a self-reported history of a physician-related diagnosis of diabetes or a history of drug treatment of diabetes. Using the Center for Epidemiologic Studies Depression Scale (CES-D) Scoresheet, we assessed the presence of depression. We applied the State-Trait Anxiety Inventory (STAI) to determine trait and state anxiety.

Anemia was defined by a hemoglobin concentration of less than 140 g/L in men and of less than 130 g/L in women (definition #1), and it was defined by a hemoglobin concentration of less than 130 g/L in men and of less than 120 g/L in women (definition #2). The reason to use two definitions of anemia was that anemia has not yet been generally defined in a consensus meeting or corresponding article. While the second anemia definition applied in our study accorded with the WHO (World Health Organization) definition of anemia, the first definition was based on the several previous population-based studies which addressed the prevalence of anemia and which used different definitions of anemia [1–5, 8, 9]. Anemia was classified into mild anemia (definition #1: hemoglobin concentration of 139 g/L to 110 g/L in men and of 129 g/L to 110 g/L in women; and definition #2: hemoglobin concentration of 129 g/L to 110 g/L in men and of 119 g/L to 110 g/L in women), and into moderate anemia (both definitions #1 and #2) with a hemoglobin concentration ranging between less than 110 g/L and 80 g/L in both gender. Severe anemia was defined by a hemoglobin concentration of less than 80 g/L.

We used a commercially available statistical software program (Statistical Package for Social Science, SPSS, version 25.0; IBM-SPSS Inc., Chicago, USA) for the statistical analysis. In a first step, we determined the mean hemoglobin concentration, expressed as mean ± standard deviation, and the mean prevalence of anemia, presented as mean and its 95% confidence intervals (CI). In a second step, we searched for associations in univariate analysis between the prevalence of anemia and other parameters. In a third step, we conducted a multivariable binary regression analysis with the prevalence of anemia as dependent variable and as independent variables all those parameters which were significantly associated with the prevalence of anemia in the univariate analysis. Finally, we assessed associations between the blood hemoglobin concentration and other parameters in a multivariable linear regression analysis. Odds ratios (OR) and their 95% CIs, the standardized regression coefficient beta and the non-standardized regression coefficient B were calculated. All *P*-values were two-sided and considered statistically significant when the values were less than 0.05.

## Results

Out of 5889 individuals primarily participating in the Ural Eye and Medical Study, the present study included 5864 (99.4%) individuals with biochemical blood examinations. The mean age of the study population (2559 (43.6%) men) was 59.0 ± 10.7 years (Table 1). The composition of the study population with respect to gender and age corresponded to the gender and age distribution in the Russian population according to the most recent census carried out in 2010 [9]. It showed two constrictions for the birth year groups from 1940 to 1946 and for the birth year groups from 1962 to 1970, directly and indirectly caused by the consequences of World War II.

**Table 1:** Prevalence (%) of any anemia (using two defintiions: defined as a hemoglobin concentration < 140 g/ in men and < 130 g/L in women, or defined as a hemoglobin concentration < 130 g/ in men and < 120 g/L in women), mild anemia (using two defintiions: defined as a hemoglobin concentration < 140 g/L and > 110 g/L in men; < 130 g/L and > 110 g/L in women, or defined as a hemoglobin concentration < 130 g/L and > 110 g/L in men; < 120 g/L and > 110 g/L in women) and moderate anemia (hemoglobin concentration < 110 g/L and > 80 g/L) in the Ural Eye and Medical Study, stratified by sex and age

Age Group (Years)	*n*	Any Anemia (Definition “1”; Hemoglobin Con-centration < 140 g/ in Men and < 130 g/L in Women) (%)	95% Con-fidence Intervals	Any Anemia (Definition “1”; Hemoglobin Concentration < 130 g/ in Men and < 120 g/L in Women) (%)	95% Con-fidence Intervals	Mild Anemia (Definition “1”; Hemoglobin Con-centration < 140 g/L and > 110 g/L in Men; < 130 g/L and > 110 g/L in Women) (%)	95% Con-fidence Intervals	Mild Anemia (Definition “2”; Hemoglobin Concentration < 130 g/L and > 110 g/L in Men; < 120 g/L and > 110 g/L in Women) (%)	95% Con-fidence Intervals	Moderate Anemia (Hemo-globin Concen-tration < 110 g/L and > 80 g/L) (%)	95% Confidence Intervals
Men	2559										
40–44	213	12.2	7.8, 16.6	4.2	1.5, 7.0	11.7	7.4, 16.1	3.8	1.2, 6.3	0.5	−0.5, 1.4
45–49	355	13.5	10.0, 17.1	4.5	2.3, 6.7	13.5	10.0, 17.1	4.5	2.3, 6.7	0.0	
50–54	437	17.4	13.8, 21.0	6.0	3.7, 8.2	16.0	12.6, 19.5	4.6	2.6, 6.5	1.4	0.3, 2.5
55–59	483	17.6	14.2, 21.0	5.6	3.5, 7.7	17.0	13.6, 20.3	5.0	3.0, 6.9	6.2	0.0, 1.3
60–64	403	15.1	11.6, 18.7	4.2	2.3, 6.2	15.1	11.6, 18.7	4.2	2.3, 6.2	0.0	
65–69	291	17.5	13.1, 21.9	7.6	4.5, 10.6	16.5	12.2, 20.8	6.5	3.7, 9.4	1.0	0.0, 2.2
70–74	137	27.0	19.5, 34.5	3.7	0.5, 6.8	26.3	18.8, 33.7	2.9	0.1, 5.8	0.7	0.0, 2.2
75–79	164	31.7	24.5, 38.9	9.8	5.2, 14.4	29.9	22.8, 37.0	7.9	3.8, 12.1	1.8	0.0, 3.9
80+	76	44.7	33.3, 56.2	18.4	9.5, 27.3	44.7	33.3, 56.2	18.4	9.5, 27.3	0.0	
Total, Men	2559	18.4	16.9, 19.9	5.9	5.0, 6.9	17.7	16.2, 19.2	5.3	4.4, 6.1	0.7	0.4, 1.0
Women	3305										
40–44	281	34.5	28.9, 40.1	15.3	11.1, 19.5	26.7	21.5, 31.9	7.5	4.4, 10.6	7.8	4.7, 11.0
45–49	387	37.7	32.9, 42.6	18.1	14.2, 21.9	29.1	24.7, 33.8	9.6	6.6, 12.5	8.5	5.7, 11.3
50–54	491	21.6	17.9, 25.2	5.9	3.8, 8.0	17.1	13.8, 20.5	1.4	0.4, 2.5	4.5	2.6, 6.3
55–59	549	20.8	17.4, 24.2	5.1	3.3, 7.0	18.6	15.3, 21.8	2.9	1.5, 4.3	2.2	1.0, 3.4
60–64	513	23.4	19.7, 27.1	5.9	3.8, 7.9	21.8	18.3, 25.4	4.3	2.5, 6.1	1.6	0.5, 2.6
65–69	496	28.2	24.3, 32.2	7.1	4.8, 9.3	24.4	20.1, 28.2	3.2	1.7, 4.8	3.8	2.1, 5.5
70–74	220	22.3	16.7, 27.8	5.9	2.8, 9.1	19.1	13.9, 24.3	2.7	0.6, 4.9	3.2	0.8, 5.5
75–79	245	38.0	31.8, 44.1	12.7	8.5, 16.9	33.1	27.1, 39.0	7.8	4.4, 11.1	4.9	2.2, 7.6
80+	123	40.7	31.9, 49.5	17.9	11.0, 24.8	30.1	21.9, 38.3	7.3	2.7, 12.0	10.6	5.1, 16.1
Total, Women	3305	27.7	26.2, 29.2	9.1	8.1, 10.1	23.2	21.8, 24.7	4.6	3.9, 5.4	4.5	3.8, 5.2

The group of individuals with information about the hemoglobin concentration and the group of subjects without blood examination data did not differ significantly in age (59.0 ± 10.7 years versus 61.1 ± 12.9 years; *P* = 0.33), gender (2259 (43.6%) men / 3305 (56.4%) women versus 21 (60.0%) men / 14 (40.0%) women; *P* = 0.06), and level of education (*P* = 0.98).

**Fig. 1:**
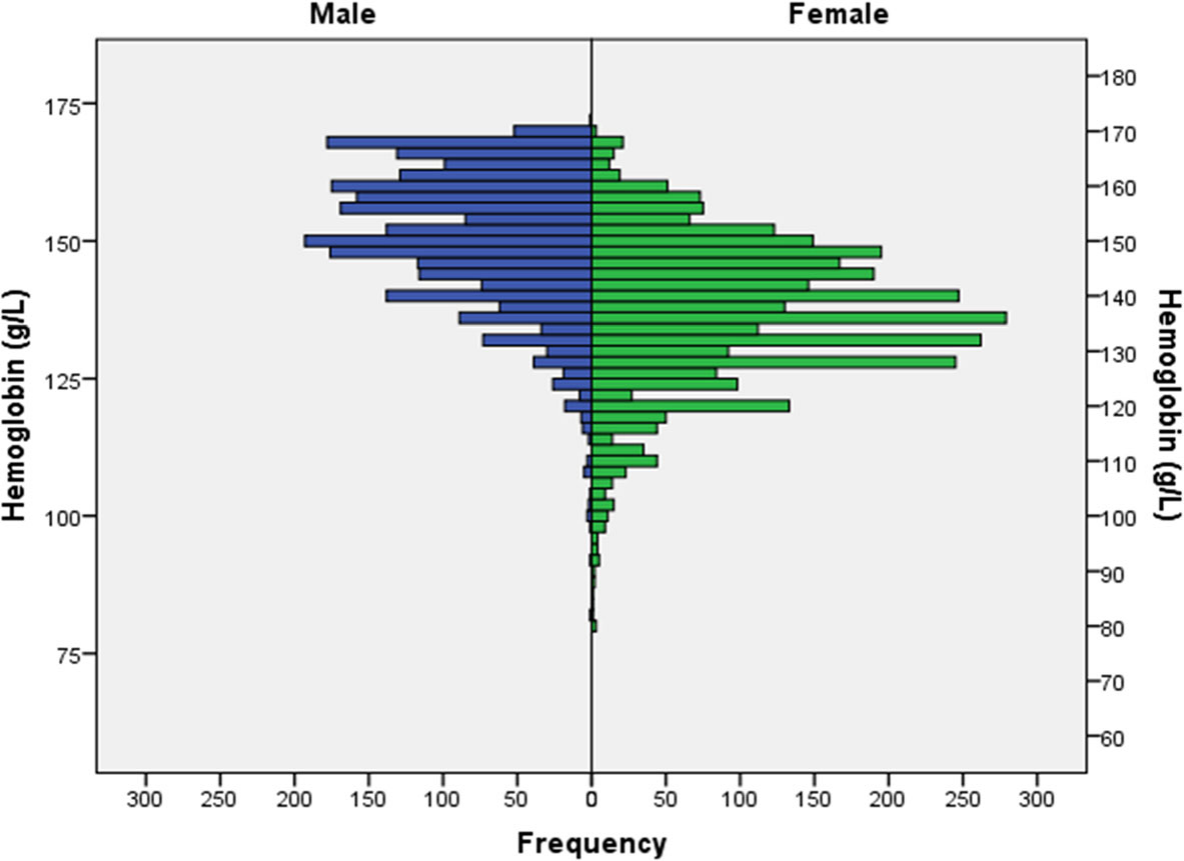
Histogram showing the distribution of the blood hemoglobin concentration stratified by gender in the Ural Eye and Medical Study

The mean hemoglobin concentration was 142.6 ± 14.8 g/L (median: 144 g/L; range: 80–171 g/L) (Fig. 1). It was significantly higher in men than in women (150.4 ± 12.6 g/L versus 136.6 ± 13.6 g/L; *P* < 0.001). Applying definition #1, anemia as a whole was detected in 1385 individuals (23.6%; 95%CI: 22.5, 24.7), and mild anemia was present in 1220 individuals (20.8%; 95%CI: 19.8, 21.8). The age-standardized prevalence rates based on the WHO world standard population aged 40+ years were 23,5% for anemia as a whole (definition #1) and 20,2% for mild anemia (definition #1). Using definition #2, anemia as a whole was found in 453 individuals (7.7%; 95%CI: 7.0, 8.4), and mild anemia was present in 288 individuals (4.9%; 95%CI: 4.4, 5.5). The age-standardized prevalence rates based on the WHO world standard population aged 40+ years were 8.2% for anemia as a whole (definition #2) and 5.1% for mild anemia (definition #2). Among the 288 individuals with mild anemia, 35 (12.2%) participants reported about the presence of an iron-deficiency anemia. Moderate anemia (both definitions #1 and #2) was diagnosed for 165 individuals (2.8%; 95%CI: 2.4, 3.2), out of whom 38 (23%) participants reported about the presence of an iron-deficiency anemia. The age-standardized prevalence rate for moderate anemia as based on the WHO world standard population aged 40+ years was 3.2%. None of study participants had severe anemia.

Within the Russian group (*n* = 1182; 506 (42.8%) men) with a mean age of 60.1 ± 11.1 years, the prevalence of anemia (definition #1) as a whole was 266 / 1182 (22.5%; 95%CI: 20.1, 24.9), the prevalence of mild anemia (definition #1) was 230 / 1182 (19.5%; 95%CI: 17.2, 21.7), and the prevalence of moderate anemia was (36 / 1182; 3.0%; 95%CI: 2.1, 4.0) (Table 2). Applying the anemia definition #2, the anemia prevalence as a whole was 79 / 1182 (6.7%; 95%CI: 5.3, 8.1), and the prevalence of mild anemia was 43 / 1182 (3.6%; 95%CI: 2.6, 4.7). The prevalences of anemia as a whole (definition #1), of mild anemia (definition #1) and moderate anemia did not differ significantly between the Russian group and the non-Russian group (*P* = 0.44, *P* = 0.27, and *P* = 0.55, respectively). In a similar manner, the prevalence of anemia as a whole (definition #2) and of mild anemia (definition #2) did not vary significantly between the Russian group and the non-Russian group (*P* = 0.26 and *P* = 0.052, resp.). As a corollary, the hemoglobin concentration did not vary significantly (*P* = 0.06; beta: 0.02) between both ethnic groups after adjusting for gender.

**Table 2:** Associations of the prevalence of anemia (defined as a hemoglobin concentration < 140 g/ in men and < 130 g/L in women) in the Ural Eye and Medical Study, after adjusting for gender and age

Parameter	*P*-Value	Odds Ratio	95% Confidence Intervals
Urban / rural region of habitation	0.38		
Family status: Married / Unmarried / Divorced / Widowed / Missing	0.008	1.08	1.02, 1.14
Family status: Married versus any other status	0.01	0.83	0.72, 0.96
Family type: Joint (three generations) / nuclear (two generations) / single / family of 2 people	0.20		
Religion: Muslim / Christian / Other	0.17		
Religion: Muslim / any other religion	0.07	1.12	0.99, 1.28
Ethnicity: Russian / any other ethnicity	0.16		
Body height (cm)	0.13		
Body weight (kg)	< 0.001	0.99	0.98, 0.99
Body mass index (kg/m^2^)	< 0.001	0.97	0.96, 0.98
Waist circumference (cm)	< 0.001	0.99	0.98, 0.99
Hip circumference cm)	0.001	0.99	0.99, 0.997
Waist-Hip-Ratio	< 0.001	0.18	0.08, 0.38
Socioeconomic parameters			
Level of education	0.11		
Monthly Income (Below poverty line / average / above average / high)	0.04	0.88	0.77, 0.99
Own ownership of house (yes / no)	0.08	0.87	0.74, 1.02
Own ownership of refrigerator (yes / no)	0.10	0.45	0.18, 1.15
Own ownership of second house (yes / no)	0.007	0.72	0.58, 0.91
Own ownership of telephone (yes / no)	0.79		
Own ownership of smartphone (yes / no)	0.39		
Own ownership of television set (yes / no)	0.60		
Own ownership of car (yes / no)	0.001	0.72	0.60, 0.87
Own ownership of two-wheeler (yes / no)	0.66		
Own ownership of tractor (yes / no)	0.10	1.47	0.93, 2.31
Own ownership of bullock cart (yes / no)	0.62		
Own ownership of computer (yes / no)	< 0.001	0.71	0.59, 0.86
Physical activity			
How long is your usual work day? (Minutes)	0.79		
Does your work involve mostly sitting or standing with less than 10 min of walking at a time? (Yes / No)	0.97		
Does your work involve physically vigorous activity (like heavy lifting or digging) or physically moderate intensity activity (like brisk walking or carrying light loads) (Yes / No)	0.38		
How many days a week do you do such physically vigorous activity during work? (Yes / No)	0.67		
On a usual day how much time do you spend on such physically vigorous work during work? (Minutes)	0.96		
Does your work involve physically moderate-intensive activity, like brisk walking or carrying light loads for at least 10 min at a time?	0.96		
In a typical week, on how many days do you do physically moderate to intensive activities as part of your work?	0.10		
Per mean day including all days of the week, how much time do you spend with physically moderate to intensive activities as part of your work?	0.59		
Do you walk or use a bicycle (pedal cycle) for at least 10 min continuously to get to and from places?	0.52		
In a typical week, on how many days do you walk or bicycle for at least 10 min to go to and from places?	0.44		
How much time do you spend walking or bicycling for travel in a day?	0.54		
Does your recreation, sport or leisure time involve mostly sitting, reclining or standing activities, with no physical activity lasting more than 10 min at a time?	0.53		
In your leisure time, do you do any physically vigorous activities like running, strenuous sports or weight lifting for at least 10 min at a time?	0.52		
If yes, In a typical week, on how many days do you do physically vigorous activities as part of your leisure time?	0.62		
How much time do you spend on physically vigorous activities as part of your leisure time on a typical day?	0.28		
In your leisure time, do you do any moderate intensity activities like brisk walking, cycling or swimming for at least 10 min at a time?	0.97		
In a typical week, on how many days do you do physically moderate to intensive activities as part of your leisure time?	0.76		
How much time do you spend on physically moderate to intensive activities per day of week during your leisure time? (Minutes)	0.63		
Over the past 7 days, how much time did you spend sitting or reclining on a typical day?	0.15		
History of diseases			
History of arterial hypertension	0.002	0.81	0.71, 0.93
History of arthritis	0.11		
History of low back pain	0.11		
History of thoracic spine pain	0.34		
History of neck pain	0.94		
History of headache	0.53		
History of therapy of hyperlipidemia	0.55		
History of cancer	0.94		
History of cardiovascular disorders including stroke	0.91		
History of dementia	0.67		
History of diabetes mellitus	0.88		
History of diarrhea	0.35		
History of bone fracture	0.18		
History of heart attack	0.33		
History of iron-deficiency anemia	< 0.001	3.22	2.53, 4.10
History of low blood pressure and hospital admittance	0.12		
History of osteoarthritis	0.65		
History of skin disease	0.18		
History of use of steroids	0.55		
History of thyreopathy	0.56		
History of tumbling	0.08	0.87	0.74, 1.02
History of unconsciousness	0.42		
Age of the last menstrual bleeding (years)	0.92		
Age of last regular menstrual bleeding (years)	0.67		
History of menopause	< 0.001	0.48	0.38, 0.62
Blood concentrations (mmol/L) of:			
Alanine aminotransferase (IU/L)	0.003	0.99	0.99, 0.997
Aspartate aminotransferase (IU/L)	0.02	0.99	0.99, 0.999
Bilirubin, total (μmol/L)	< 0.001	0.99	0.99
High-density lipoproteins (mmol/L)	0.88		
Low-density lipoproteins (mmol/L)	0.02	0.94	0.89, 0.99
Triglycerides (mmol/L)	< 0.001	0.80	0.72, 0.88
Cholesterol (mmol/L)	< 0.001	0.91	0.88, 0.95
C-reactive protein (mg/L)	0.46		
Rheumatoid factor (IU/mL)	0.001	1.10	1.04, 1.17
Erythrocyte sedimentation rate (mm / hour)	< 0.001	1.07	1.06, 1.08
Glucose (mmol/L)	0.28		
Creatinine (μmol/L)	0.001	1.004	1.002, 1.007
Urea (mmol/L)	0.001	1.08	1.03, 1.12
Residual nitrogen (g/L)	0.09	2.06	0.89, 4.76
Total protein (g/L)	0.004	0.99	0.98, 0.996
International normalized ratio (INR)	0.09	1.44	0.95, 2.19
Blood clotting time (minutes)	< 0.001	9.35	7.90, 11.1
Prothrombin time (%)	0.05	0.99	0.99, 1.00
Erythrocytes (10^6^ cells / μL)	< 0.001	0.000	0.000, 0.001
Leukocytes (10^9^ cells / L)	< 0.001	0.89	0.86, 0.94
Rod-core granulocyte (% of leukocytes)	< 0.001	1.45	1.39, 1.51
Segment nuclear granulocyte (% of leukocytes)	< 0.001	0.95	0.94, 0.95
Eosinophil granulocytes (% of leukocytes)	< 0.001	1.22	1.14, 1.29
Lymphocytes (% of leukocytes)	< 0.001	1.02	1.02, 1.03
Monocytes (% of leukocytes)	< 0.001	1.05	1.03, 1.08
Blood pressure, systolic (mmHg)	0.01	0.996	0.993, 0.999
Blood pressure, diastolic (mmHg)	< 0.001	0.98	0.98, 0.99
Blood pressure, mean (mmHg)	0.003	0.99	0.98, 0.996
Ankle-brachial index, right side	0.17		
Ankle-brachial index, left side	0.07	1.51	0.97, 2.22
Medical Doctor seen within the last year	0.91		
Diet			
Vegetarian diet / mixed diet	0.74		
Number of meals per day	0.51		
In a week how many days do you eat fruits?	0.46		
How many servings of fruit do you take on one of those days (g)	0.77		
In a week how many days do you eat vegetables?	0.35		
How many servings of vegetables do you eat on one of those days (gram)?	0.52		
Type of oil used for cooking: vegetable oil / non-vegetable oil	0.63		
Food containing whole grains (Yes / No)	0.52		
Salt consumed per day (g)	0.71		
Degree of processing of meat (weak / medium / well done)	0.47		
Smoking			
Do you currently smoke any tobacco products? (yes)	0.39		
Do you smoke daily? (yes / no)	0.40		
How old were you when you first started smoking? (years)	0.95		
Have you stopped smoking? (yes / no)	0.81		
How many cigarettes do smoke each day? (0 / ≤10 / 11–20 / 21–30 / > 30)	0.55		
Package years (package = 20 cigarettes)	0.51		
Do you use smokeless tobacco (snuff, chewing tobacco)?	0.99		
If yes, daily? (yes / no)	0.66		
How much time after awakening do you smoke the first cigarette of the day? (< 5 min / 6–30 min. / 31–60 min. / > 60 min.)	0.009	0.76	0.62, 0.93
Difficult to refrain from smoking in forbidden places? (yes / no)	0.99		
Do you smoke more frequently during the first hours after waking than during the rest of the day? (yes / no)	0.93		
Do you smoke when you ill? (yes / no)	0.34		
Alcohol			
Alcohol consumed such as beer, whisky, rum, gin brandy or other local products? (yes / no)	0.13		
Age when you first started to drink alcohol?	0.24		
Did you stop drinking alcohol and are you still completely abstinent?	0.87		
Age when you stopped drinking alcohol?	0.82		
How many alcoholic drinks do you have on a typical day when you are drinking)	0.52		
How often do you have 6 or more drinks on one occasion? (never / rarely / sometimes / often / cannot say)	0.49		
How often during the last year have you found that you were not able to stop drinking once you had started? (never / rarely / sometimes / often / cannot say)	0.003	1.63	1.19, 2.25
How often during the last year have you failed to do what was normally expected from you because of drinking? (never / rarely / sometimes / often / cannot say)	0.02	1.55	1.08, 2.24
How often during the last year have you needed a first drink in the morning to get yourself going after a heavy drinking? (never / rarely / sometimes / often / cannot say)	0.16		
How often during the last year have you had a feeling of guilt or remorse after drinking? (never / rarely / sometimes / often / cannot say)	0.01	1.45	1.10, 1.92
How often during the last year have you been unable to remember what happened the last night? (never / rarely / sometimes / often / cannot say)	0.26		
Have you or someone else has been injured as a result of your drinking?	0.19		
Has a relative, friend or a doctor or another health worker been concerned about your drinking or suggested you to drink less?	0.22		
Hearing loss			
Do you experience the hearing loss (no / sometimes / yes)	0.002	1.06	1.02, 1.10
Does a hearing problem cause you to feel embarrassed when meeting new people? (no / sometimes / yes)	0.02	1.06	1.01, 1.12
Does a hearing problem cause you to feel frustrated when talking to members of your family? (no / sometimes / yes)	0.07	1.05	1.00, 1.10
Do you have difficulties in hearing when someone speaks in a whisper tone? (no / sometimes / yes)	0.04	1.05	1.002, 1.10
Do you feel handicapped by a hearing problem? (no / sometimes / yes)	0.07	1.06	0.996, 1.12
Does a hearing problem cause you difficulties when visiting friends, relatives, or neighbors?	0.09	1.05	0.99, 1.11
Does a hearing problem cause you to attend religious services less often than you would like?	0.04	1.07	1.003, 1.14
Does a hearing problem cause you to have arguments with family members?	0.29		
Does a hearing problem cause you to have difficulties when listening to TV or radio?	0.08	1.05	1.00, 1.10
Do you feel any difficulty with your hearing limits hampering your personal or social life?	0.04	1.07	1.003, 1.13
Does a hearing problem cause you difficulties when in a restaurant with relatives or friends?	0.04	1.07	1.003, 1.14
Hearing Loss Total Score	0.02	1.01	1.001, 1.01
Webers test (> right eye / > left eye / equal)	0.02	0.80	0.67, 0.96
Rinne test right ear (positive)	0.62		
Rinne test left ear (positive)	0.56		
Depression			
I was bothered by things that usually don’t bother me.	0.22		
I did not feel like eating, my appetite was poor	0.045	1.15	1.003, 1.31
I felt that I could not shake off the blues, even with the help from family and friends	0.61		
I felt that I was just as good as other people	0.18		
I had trouble keeping my mind on what I was doing	0.28		
I felt depressed	0.91		
I felt that everything I did was an effort	0.41		
I felt hopeful about the future	0.36		
I thought my life had been a failure	0.80		
I felt fearful	0.49		
My sleep was restless	0.18		
I was happy	0.52		
I talked less than usual	0.22		
I felt lonely	0.75		
People were unfriendly	0.05	1.17	1.00, 1.36
I enjoyed life	0.45		
I had crying spells	0.24		
I felt sad	0.97		
I felt that people dislike me	0.92		
I could not get “going”	0.06	1.15	0.9971.33
Depression score (adapted)	0.46		
State-Trait Anxiety Inventory (STAI)			
I feel pleasant	0.40		
I tire quickly	0.85		
I feel like crying	0.35		
I wish I could be as happy as others seem to be	0.91		
I am losing out on things because I can’t make up my mind soon enough	0.04	1.16	1.01, 1.34
I feel rested	0.20		
I am calm, cool and collected	0.83		
I feel that difficulties are piling up so that I can’t overcome them	0.09	1.13	0.98, 1.30
I worry too much over something that really doesn’t matter	0.33		
I am happy	0.96		
I am inclined to take things hard	0.30		
I lack self-confidence	0.77		
I feel safe	0.43		
I try to avoid facing a crises or difficulty	0.23		
I feel blue	0.20		
I am content	0.26		
Some unimportant thoughts run through my mind and bother me	0.11		
I take disappointments so keenly that I can’t put them out of my mind	0.83		
I am a steady person	0.68		
I get in a state of tension or turmoil as I think over my recent concerns and interests	0.42		
State-Trait Anxiety Inventory (STAI) Score (adapted)	0.73		
I attempted suicide due to financial reasons then	0.51		
Have you thought of committing suicide in the last 6 months or earlier?	0.46		
I thоught of suicide	0.31		
I thоught of suicide due to financial reasons then	0.67		
Dynamometry			
Manual dynamometry, right hand (dekaNewton)	< 0.001	0.98	0.97, 0.99
Manual dynamometry, left hand (dekaNewton)	< 0.001	0.98	0.97, 0.99

**Fig. 2:**
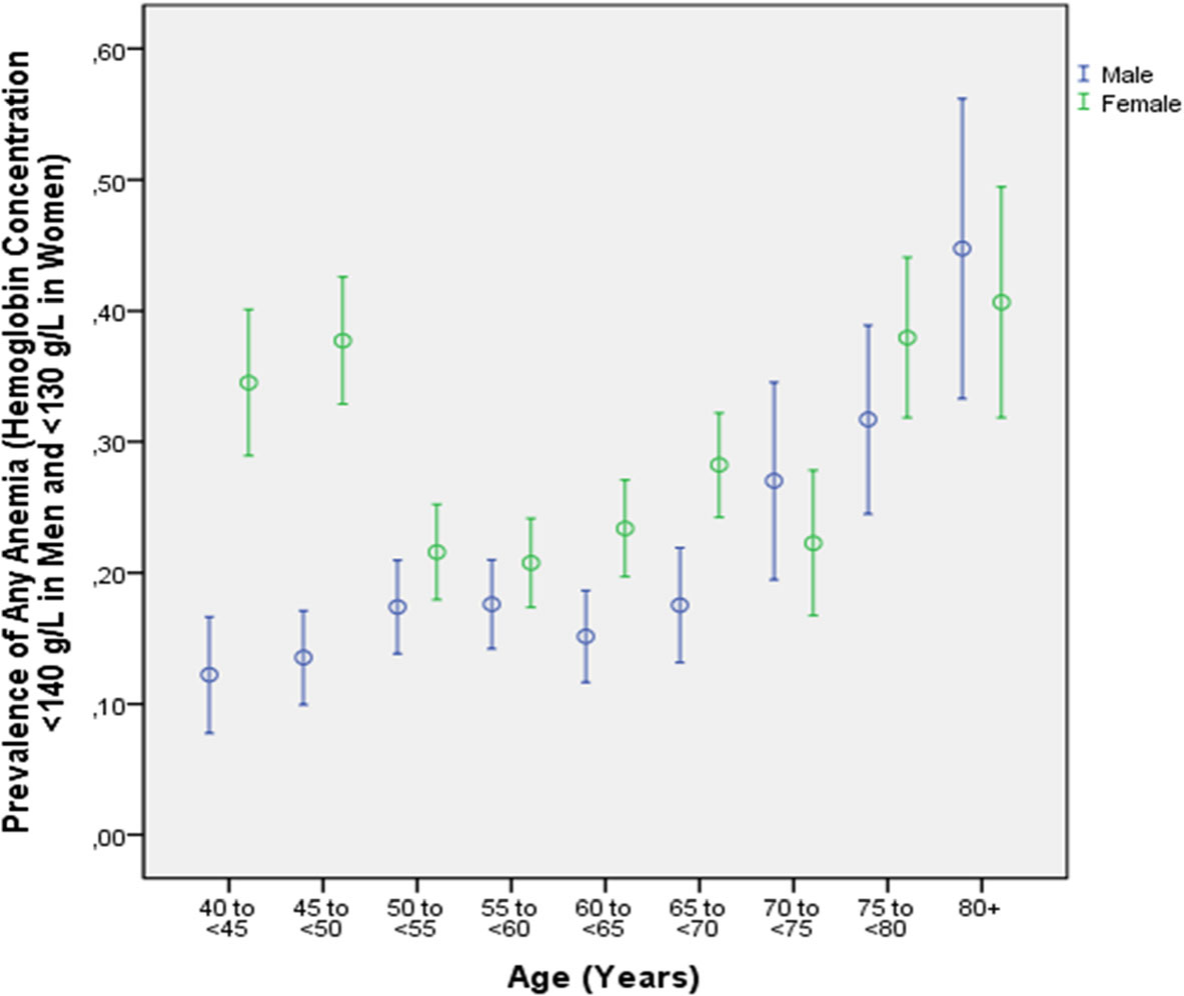
Graph showing the prevalence of anemia stratified by age and gender in the Ural Eye and Medical Study

In univariate analysis, a higher prevalence of anemia (definition #1) was associated with older age (*P* < 0.001) and female gender (*P* < 0.001) (Fig. 2). For exploring any other associations, we adjusted the analysis for age and gender. After adjusting for age and gender, a higher prevalence of anemia was associated with lower prevalence of married family status (*P* = 0.01), higher prevalence of Muslima religion (*P* = 0.07), lower body weight (*P* < 0.001) and lower body mass index (*P* < 0.001), shorter waist circumference (*P* < 0.001), shorter hip circumference (*P* = 0.001), and lower waist-to-hip circumference ratio (*P* < 0.001), lower monthly income (*P* = 0.04), lower frequency of an ownership of a car (*P* = 0.001), lower prevalence of a history of arterial hypertension (*P* = 0.002), tumbling (*P* = 0.08) and menopause (*P* < 0.001), higher prevalence of a history of iron-deficiency anemia (*P* < 0.001), lower blood concentration of alanine transferase (*P* = 0.003), aspartate aminotransferase (*P* = 0.02), bilirubin (*P* < 0.001), low-density lipoproteins (*P* = 0.02), triglycerides (*P* < 0.001), cholesterol (*P* < 0.001), higher blood concentration of the rheumatoid factor (*P* = 0.001), creatinine (*P* = 0.001), urea (*P* = 0.001), residual nitrogen (*P* = 0.09), faster erythrocyte sedimentation rate (*P* < 0.001), longer blood clotting time (*P* < 0.001), lower prothrombin time (*P* = 0.05), lower blood count of leukocytes (*P* < 0.001), any type of granulocytes (*P* < 0.001), lymphocytes and monocytes (*P* < 0.001), lower systolic (*P* = 0.01) and diastolic (*P* < 0.001) blood pressure, higher ankle-brachial index (*P* = 0.07), higher prevalence of not able to stop drinking alcohol once started (*P* = 0.003), of failing to do what was normally expected due to alcohol consumption (*P* = 0.02) and of feeling guilty after drinking (*P* = 0.01), higher total hearing loss score (*P* = 0.02), and lower manual dynamometric force (*P* < 0.001) (Table 3).

**Table 3:** Associations (multivariate analysis) of the prevalence of anemia (defined as a hemoglobin concentration < 140 g/ in men and < 130 g/L in women) in the Ural Eye and Medical Study

Parameter	*P*-Value	Odds Ratio	95% Confidence Interval
Sex (Men / Women)	< 0.001	0.52	0.41, 0.65
Waist-Hip Circumference Ratio	0.002	0.16	0.05, 0.51
Ownership of car	0.005	0.74	0.61, 0.92
Blood Concentration of Bilirubin (μmol/L)	< 0.001	0.98	0.97, 0.99
Blood Concentration of Triglycerides (mmol/L)	0.003	0.77	0.65, 0.91
Erythrocyte Sedimentation Rate (mm / hour)	< 0.001	1.07	1.06, 1.08
Blood Clotting Time (min)	< 0.001	8.65	6.68, 11.2
Leukoycte Count (10^9^ cells/L)	0.03	0.93	0.87, 0.99

The multivariable regression analysis included the prevalence of anemia (definition #1) as dependent variable and as independent variables all those parameters which were significantly associated with the prevalence of anemia in the univariate analysis. Due to collinearity we first dropped the parameters of body weight, and hip and waist circumference. Due to a lack of statistical significance, we dropped the parameters of alcohol consumption-related parameters (“feeling guilty after drinking alcohol” (*P*=0.73); “failing to do what was normally expected due to alcohol consumption” (*P* = 0.89); “not able to stop drinking alcohol once started” (*P* = 0.10)), religion (*P* = 0.97), blood concentrations of rheumatoid factor (*P* = 0.99), low-density lipoproteins (*P* = 0.79), urea (*P* = 0.83), alanine transferase (*P* = 0.95), aspartate aminotransferase (*P* = 0.39), creatinine (*P* = 0.45) and prothrombin time (*P* = 0.49), family status (married versus non-married) (*P* = 0.43), history of tumbling (*P* = 0.78) and arterial hypertension (*P* = 0.58), manual dynamometric force (*P* = 0.42), count of lymphocytes (*P* = 0.21) and monocytes (*P* = 0.24), ankle-brachial index (*P* = 0.34), systolic blood pressure (*P* = 0.30), body mass index (*P* = 0.28), self-reported income (*P* = 0.19), blood residual nitrogen (*P* = 0.16), hearing loss score (*P* = 0.17), blood concentration of cholesterol (*P* = 0.11), diastolic blood pressure (*P* = 0.08) and age (*P* = 0.33). In the final model, a higher prevalence of anemia (definition #1) was associated with male gender (*P* < 0.001), lower waist-hip circumference ratio (*P* = 0.002), lower prevalence of an ownership of a car (*P* = 0.005), lower blood concentrations of bilirubin (*P* < 0.001) and triglycerides (*P* = 0.003), higher erythrocyte sedimentation rate (*P* < 0.001), longer blood clotting time (*P* < 0.001) and lower count of leukocytes (*P* = 0.03) (Table 4). If the parameter of car ownership was replaced by the parameter of level of education, lower educational level was significantly associated with higher anemia prevalence (*P* = 0.001; OR: 0.93; 95%CI: 0.89, 0.97). There was a tendency for higher anemia prevalence in the urban versus rural region of habitation (*P* = 0.10; OR: 0.84; 95%CI: 0.68, 1.03).

**Table 4:** Associations (multivariate analysis) of the prevalence of any anemia (defined as a hemoglobin concentration < 130 g/ in men and < 120 g/L in women) in the Ural Eye and Medical Study

Parameter	*P*-Value	Odds Ratio	95% Confidence Interval
Age (Years)	0.045	0.98	0.97, 1.00
Sex (Men / Women)	< 0.001	0.43	0.30, 0.60
Waist-Hip Circumference Ratio	0.01	0.10	0.02, 0.63
Ownership of car	0.006	0.63	0.45, 0.88
Blood Concentration of Bilirubin (μmol/L)	0.006	0.97	0.96, 0.99
Erythrocyte Sedimentation Rate (mm / hour)	< 0.001	1.08	1.07, 1.10
Blood Clotting Time (min)	< 0.001	6.99	4.76, 10.3
Leukoycte Count (10^9^ cells/L)	0.002	0.84	0.76, 0.94

In a similar manner, a higher prevalence of anemia as defined by definition #2 was associated with male gender (*P* < 0.001), lower waist-hip circumference ratio (*P* = 0.01), lower prevalence of an ownership of a car (*P* = 0.006), lower blood concentrations of bilirubin (*P* = 0.006) and triglycerides (*P* = 0.003), higher erythrocyte sedimentation rate (*P* < 0.001), longer blood clotting time (*P* < 0.001) and lower count of leukocytes (*P* = 0.03), and additionally with younger age (*P* = 0.045) (Table 5). In women, it was significantly higher before menopause (16.6%; 95%CI: 13.5, 19.7) than after menopause (7.1%; 95%CI: 6.0, 8.1).

**Table 5:** Associations (multivariate analysis) of the prevalence of moderate anemia (defined as a hemoglobin concentration < 110 g/L and > 80 g/L) in the Ural Eye and Medical Study

Parameter	*P*-Value	Odds Ratio	95% Confidence Interval
Age (Years)	0.008	0.98	0.96, 0.99
Sex (Men / Women)	0.001	2.52	1.47, 4.33
Waist-Hip Circumference Ratio	0.058	0.13	0.02, 1.07
Erythrocyte Sedimentation Rate (mm / hour)	< 0.001	1.08	1.06, 1.09
Blood Clotting Time (min)	< 0.001	8.56	5.68, 12.9

Higher prevalence of moderate anemia was correlated with younger age (*P* = 0.008), female gender (*P* < 0.001), higher erythrocyte sedimentation rate (*P* < 0.001), longer blood clotting time (*P* < 0.001), and marginally significantly, with a lower waist-hip circumference ratio (*P* = 0.058) (Table 6). In women, it was significantly higher before menopause (8.8%; 95%CI: 6.4, 11.1) than after menopause (3.5%; 95%CI: 2.8, 4.3).

**Table 6:** Multivariate linear regression analysis of associations of the blood hemoglobin concentration in the Ural Eye and Medical Study

Parameter	*P*-Value	Standardized Regression Coefficient beta	Non-Standardized Regression Coefficient B	95% Confidence Interval
Sex (Men / Women)	< 0.001	−0.20	−5.92	−6.75, − 5.09
Waist-Hip Circumference Ratio	< 0.001	0.05	8.25	4.08, 12.4
Ownership of Car	< 0.001	0.05	1.57	0.78, 2.37
Blood Concentration of Bilirubin (μmol/L)	< 0.001	0.05	0.07	0.04, 0.10
Blood Concentration ofTriglycerides (mmol/L)	< 0.001	0.06	1.29	0.76, 1.82
Erythrocyte Sedimentation Rate (mm / hour)	< 0.001	−0.32	−0.41	−0.45, − 0.38
Blood Clotting Time (min)	< 0.001	−0.39	−10.9	−11.6, −10.2

In multivariable linear regression analysis, the blood hemoglobin concentration was associated (regression coefficient r^2^ = 0.50) with male gender (*P* < 0.001), lower waist-hip circumference ratio (*P* < 0.001), lower prevalence of an ownership of a car (*P* < 0.001), lower blood concentrations of bilirubin (*P* < 0.001) and triglycerides (*P* < 0.001), higher erythrocyte sedimentation rate (*P* < 0.001) and longer blood clotting time (*P* < 0.001) (Table 7). If the parameter of car ownership was replaced by the parameter of level of education, higher educational level was significantly associated with higher hemoglobin concentration (*P* = 0.007; beta: 0.03).

**Table 7:** Multivariable linear regression analysis of associations of the blood hemoglobin concentration in the Ural Eye and Medical Study

Parameter	*P*-Value	Standardized Regression Coefficient beta	Non-Standardized Regression Coefficient B	95% Confidence Interval
Sex (Men / Women)	< 0.001	−0.20	−5.92	−6.75, −5.09
Waist-Hip Circumference Ratio	< 0.001	0.05	8.25	4.08, 12.4
Ownership of Car	< 0.001	0.05	1.57	0.78, 2.37
Blood Concentration of Bilirubin (μmol/L)	< 0.001	0.05	0.07	0.04, 0.10
Blood Concentration ofTriglycerides (mmol/L)	< 0.001	0.06	1.29	0.76, 1.82
Erythrocyte Sedimentation Rate (mm / hour)	< 0.001	−0.32	−0.41	−0.45, − 0.38
Blood Clotting Time (min)	< 0.001	−0.39	−10.9	−11.6, −10.2

Similar results were obtained if the parameters of erythrocyte sedimentation rate and blood clotting time as factors secondary to anemia were dropped from the model,

## Discussion

In our population-based study in Russia, higher hemoglobin concentration was associated with male gender, higher waist-hip circumference ratio, higher level of education, higher blood concentrations of bilirubin and triglycerides, lower erythrocyte sedimentation rate and shorter blood clotting time. The prevalence of anemia defined by a hemoglobin concentration < 140 g/L in men and < 130 g/L in women was 23.6% (95% CI: 22.5, 24.7), and the prevalence of anemia defined by a hemoglobin concentration of < 130 g/L in men and < 120 g/L in women was 7.7% (95%CI: 7.0, 8.4). The prevalence of moderate anemia (hemoglobin concentration: 110 g/L to 80 g/L) was 2.8% (95%CI: 2.4, 3.2) and increased with younger age, female gender, higher erythrocyte sedimentation rate, longer blood clotting time, and marginally significantly, with a lower waist-hip circumference ratio. In women, it was significantly (*P* < 0.001) higher before menopause (8.8%; 95%CI: 6.4, 11.1) than after menopause (3.5%; 95%CI: 2.8,4.3) (Fig. 2). These prevalence figures for this study population in Russia were relatively low as compared with other populations. As in other populations, the prevalence of anemia was strongly associated with menopause, and to a minor degree, with a decreased lower waist-hip circumference ratio and lower socio-economic and educational background. There was a tendency for a higher anemia prevalence in the urban region of habitation (*P* = 0.10).

Anemia as a major public health problem has been addressed in numerous studies before, estimating the total number of individuals affected by anemia to be 1.62 billion people globally [10]. The prevalence of anemia ranged between 9% in high-income countries and 43% in countries with a relatively low degree of development [10]. Anemia prevalence was usually higher in pregnant women, women of reproductive age and in children than in men or elderly women. Global estimates for the prevalence of anemia were as high as 47% for children with an age of less than 5 years, 42% for pregnant women, and 30% for non-pregnant women with an age of 15 to 49 years [10]. Africa and Asia were the continents bearing the load of about 85% of the absolute anemia burden in the high-risk groups. It has been estimated that globally and per year, anemia contributed to more than 591,000 perinatal deaths and 115,000 maternal deaths [11]. The figures found for our study population from Russia show that the prevalence of anemia in Russia is similar to the anemia prevalence found in high-income countries, despite the marked difference between Russia and high-income countries in the social development index (SDI) as calculated by the Global Burden of Disease Study [8–25]. Using the WHO definition of anemia, the prevalence of anemia in our study population was 7.7% (95%CI: 7.0, 8.4). Correspondingly, none of the study participants had a severe anemia defined by a hemoglobin concentration of less than 80 mg/L. These results are in contrast to anemia prevalences found in countries with a similar SDI as Russia but which show a markedly higher anemia prevalence including severe anemia [10, 11, 13–25]. It suggests that the public health care system in Russia is better than it would be expected based on its SDI. It agrees with the finding of a relatively low unawareness rate for diabetes of 25% in the Russian population (Ural Eye and Medical Study; own data), a figure similar or even lower than the rate found in high-income countries.

The results obtained in our study on associations of anemia with other systemic parameters are in agreement with findings obtained in previous investigations. The main factor associated with anemia was female gender in the reproductive age (Fig. 2), in addition to a low waist-hip circumference ratio or low body mass index and a low socio-economic and educational background [1–3, 10, 11, 13–25].

The univariate analysis included many diverse parameters which were either the determinants of anemia or the sequels of anemia. Subsequently, the multivariate analysis consisted of causes and of consequences of anemia. To cite examples, the parameters of male gender, higher waist-hip circumference ratio, higher prevalence of car ownership and status before menopause were likely variables associated with the causation of anemia, while the variable of a higher blood concentration of bilirubin was probably a consequence of anemia. The diverse nature of the independent parameters in the multivariable analysis should therefore be taken into account when the results of the statistical analysis are discussed.

When the results of our study are discussed, its limitations should be taken into account. First, as for any population-based study, the participation rate is critical to assure the representativeness of the study population. With more than 80% of the eligible population taking part in our investigation, a pronounced bias in the inclusion of participants might have been unlikely. Second, our study population was composed of various ethnicities. While this multi-ethnic composition was typical for Southern Russia, the population of North-Western Russia and Central Russia usually shows a higher percentage of Russians. To overcome this potential limitation of our study, we assessed the prevalence of anemia in dependence of the ethnic background and found that the prevalence did not differ significantly between the Russian group and the non-Russian group. The age and gender distribution in our study population was comparable to the results of the Russian census 2010 [12]. Third, there were several definitions of anemia, and we used the anemia definition based on the blood hemoglobin concentration. It lacks, however, specificity for establishing the iron status. Measurement of the concentrations of serum ferritin and transferrin receptor in combination with determinations of the hemoglobin concentrations would have been better measures. Fourth, in the statistical analysis we did not clearly differentiate between biomarkers which were probably due to anemia and determinants of anemia.

## Conclusions

In conclusion, in this ethically mixed urban and rural Russian population aged 40+ years, the prevalence of anemia (defined by a hemoglobin concentration of < 130 g/L in men and of < 120 g/L in women) was relatively low (7.7%;95%CI:7.0,8.4), and it was similar to the anemia prevalence in high-income countries. As in other populations, the anemia prevalence was strongly associated with menopause, and to a minor degree, with a lower waist-hip circumference ratio and lower socio-economic and educational background. There was a tendency for a higher anemia prevalence in the urban region of habitation (*P* = 0.10). These data may be of interest for assessing the burden of anemia in Russia.

## Data Availability

Available on request from the corresponding author.

## Abbreviations

BMI: Body mass index
CI: Confidence intervals
Fig: Figure
GATHER: Guidelines for Accurate and Transparent Health Estimates Reporting
GBD: Global Burden of Diseases, Injuries and Risk Factors
OR: Odds ratio
SDI: Social development index
SPPS: Statistical Package for Social Science
UEMS: Ural Eye and Medical Study
YLD: Years lived with disability
